# Beyond graduation: The journey of a young family doctor in Malaysia

**DOI:** 10.51866/mol.96y

**Published:** 2025-08-09

**Authors:** Marina Antony Nicholson

**Affiliations:** 1 MBBS, MAFP, FRACGP, Klinik Kesihatan Dato’ Keramat Taman Keramat, Kuala Lumpur, Malaysia. Email: marina.antony.nicholson@gmail.com

**Keywords:** Family medicine, Primary care physicians, Professionnl development, Leadership, Education

Ten years ago, I made the decision to specialise in family medicine. My reasons were simple. I wanted to be present for patients throughout their lives - from before conception to their last breath. Most importantly, I wanted to see patients as a whole and as members of communities long before illness strikes.

In 2021, I was conferred a fellowship by the Royal Australian College of General Practitioners and a memb ershin by the Academy of Family Physicians ofMalaysia (AFPM).

After several months in practice as a family medicine specialist, I came to realise that being a young family doctor is far more than stethoscopes and prescriptions. Many senior doctors had paved the way for me, and I felt it was now my turn to contribute to the fraternity.

**Figure 1:**
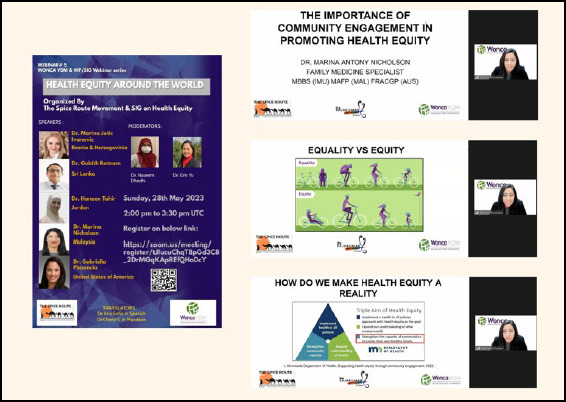
Speaking at WONCA’s Health Equity Around the World webinar.

In 2023, I ‘was appointed Promotion Lead for the Chapter of Young; Family Doctors (CoYFD) Malaysia under the AFPM. One of my first responsibilities was speaking at WONCA’s *Health Equity Around the World* webinar (Figure 1), alongside family physicians from across the globe. It was a humbling experience that allowed me to learn from the diverse experiences of the global family medicine community.

**Figure 2:**
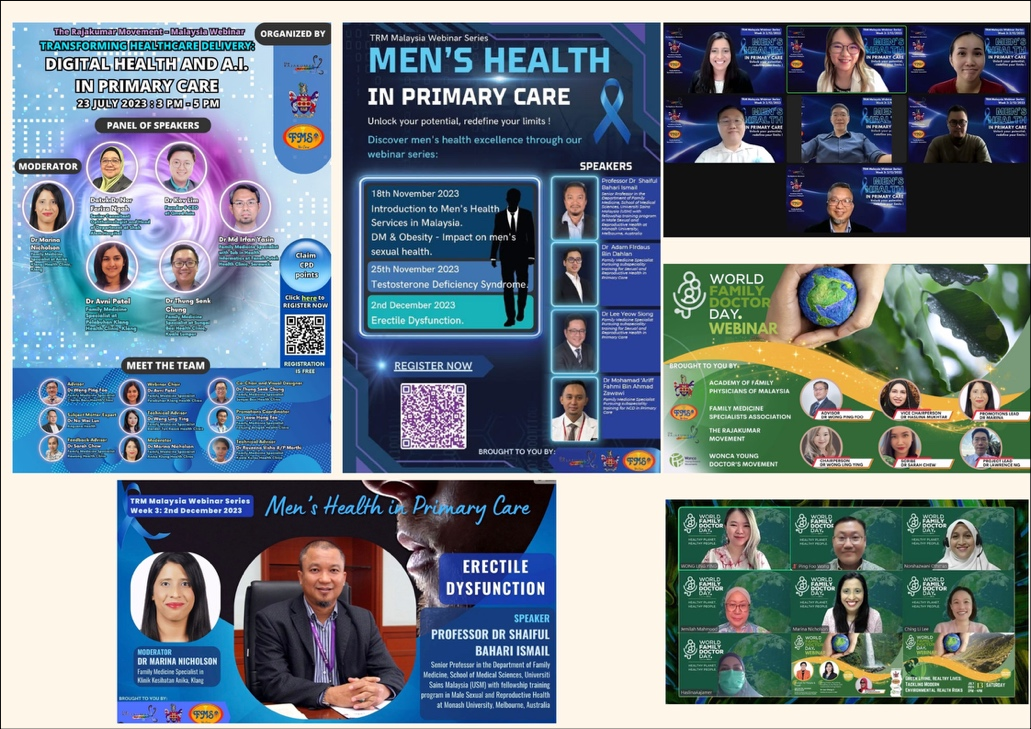
Webinars organised by the CoYFD Malaysia in collaboration with the AFPM and FMSA.

Locally, the CoYFD Malaysia has organised numerous webinars and workshops (Figure 2) in collaboration with the AFPM and Malaysian Family Medicine Specialists’ Association (FMSA). These webinars have featured many speakers who are well-known and respected ill their respective fields in Malaysia. I had the honour of myderating many of these webinars, which attracted hundreds of healthcare practitioners nationwide.

**Figure 3:**
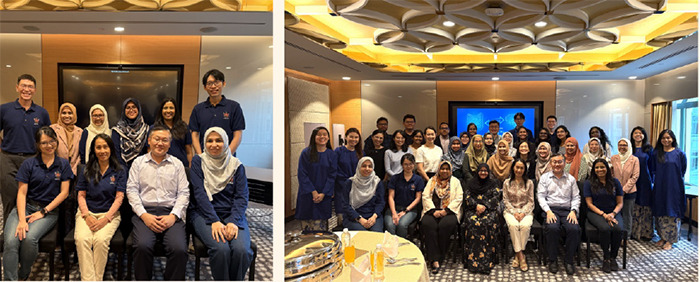
Members of the CoYFD Malaysia, together with Dr Isriyanti binti Mohd Rafae (President of the AFPM), Dr Nor Hazlin binti Talib (President of the FMSA) and Dr Wong Ping Foo (Advisor oUthe CoYFD Malaysia),duringthe networking and leadership workshop.

The CoYFD, in collaboration with the AFPM and FMSA, has also organised physical events, including a networking and leadership workshop in 2024 (Figure 3), aimed at helping young doctors develop leadership skills and effective networking strateeies. In February 2025, the CoYFD hosted a professionel image workshop led by an image consultant at the state-of-the-art Medical Academies of Malaysia. Family physicians from across Malaysia gathered to learn about enhancing their professional image.

As I approach my fifth year as a family medicine specialist, I find myself passing the baton to the next generation of young doctors. My time with the CoYFD has been truly fulfilling and I am forever grateful bor tine expenience. Through it, I have gained inualuable experience and witnessed the strength of teamwork.

**Figure 4:**
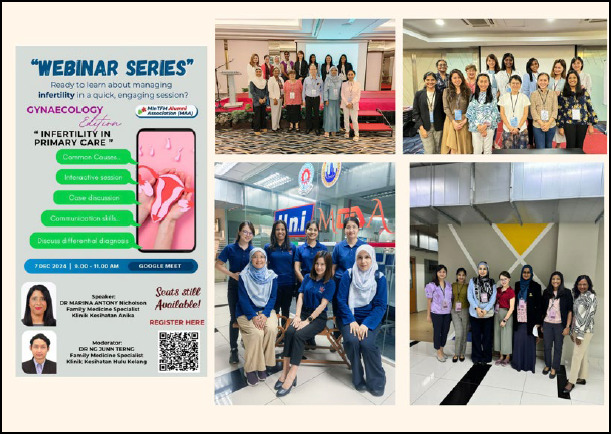
Delivering talks and facilitating workshops for candidates of the Graduate; Certificate in Family Medicine fnd Advanced Training in Family Medicine, as welt as serving as an examination coordinator for the Conioint MAFP/icFRACGP Part 1 Examinations.

However, this is just the beginning. I aim to continue learning and contributing to my peers and the field of family medicine. My passion for teaching has led me to mentorship and educational roles (Figure 4). I now serve as a mentor and examination coordinator with the AFPM.

**Figure 5:**
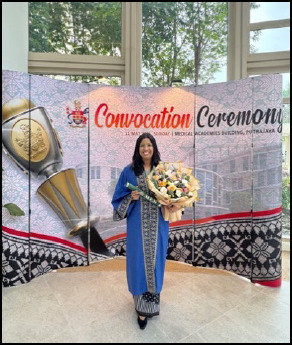
Convocation ceremony for the Certificate in Teaching in Family Medicine.

I completed the National Postgraduate Medicnl (Curriculum training id 2023 and graduated with a Certificate in Teaching in Family Medicine in May 2025 (Figure 5), marking a signidcant milestone in my teaching journey.

To me, becoming a family medicine specialist is not the end but the start of a meaningful and exciring journey ahead. We ssand on the shoulders of those who have come before us, and now it is our turn to carry the torch forward.

